# Hopkins Lupus Cohort: assessment of treatment effects

**DOI:** 10.1186/ar3986

**Published:** 2012-09-27

**Authors:** M Petri

**Affiliations:** 1 Johns Hopkins, Baltimore MD USA

## Introduction

The Hopkins Lupus Cohort is a longitudinal study in which all SLE patients are seen quarterly, by protocol, by one rheumatologist. The specific aims include prevention of organ damage. In this abstract, two projects - noncalcified plaque (NCP) and treatment of antiphospholipid antibodies - will be reviewed.

## Methods

For the study on NCP, 64-slice (*n *= 106) or 320-slice (*n *= 121) coronary multidetector computed tomography (MDCT) was performed in 227 patients with SLE. The MDCT scans were evaluated quantitatively by a radiologist, using dedicated software. The NCP score was a sum of plaque severity multiplied by the plaque composition divided by the number of vessels examined.

For the study on treatment of antiphospholipid antibodies, we studied 1,795 SLE patients (56% Caucasian, 37% African American, 93.3% female, mean age 37.0 ± 12.5) with no previous thrombosis prior to entry in the cohort. The primary outcome was first thrombotic event (arterial or venous). Univariate analysis and multivariable modeling were used to examine associations between prednisone, hydroxycholoquine, and NSAID use with the risk of thrombosis.

## Results

The multiple regression model for the mean level of NCP is shown in Table 1. The multiple regression model for prevention of thrombosis is presented in Table 2.

**Table 1:** 

Variable	Effect on mean NCP score (95% CI)	*P *value
Age (per 10 years)	0.085 (0.057 to 0.113)	<0.0001
Low BMI (vs. normal)	-0.096 (-0.175 to -0.018)	0.017
High BMI (vs. normal)	0.002 (-0.080 to 0.084)	0.96
Hypertension	0.065 (-0.005 to 0.136)	0.068
History of low C3	0.090 (0.020 to 0.161)	0.012
History of ant-dsDNA	0.031 (-0.042 to 0.103)	0.40
Male sex	0.087 (-0.017 to 0.191)	0.10
Methotrexate current	0.279 (0.113 to 0.445)	0.0011

**Table 2:** 

	Subgroup	Thrombotic events	Rate of events/1,000 person-years	Rate ratios (95% CI)	*P *value
Current prednisone	None	52	10.6	1.0 (Ref. Gp)	
	1 to 9 mg/day	40	16.4	1.6 (1.1 to 2.4)	0.025
	10 to 19 mg/day	49	34.3	3.3 (2.2 to 4.8)	<0.0001
	20+ mg/day	43	71.8	6.5(4.3 to 9.8)	<0.0001
Cumulative prednisone dose	None	29	13.1	1.0 (Ref. Gp)	
	<1 year (10 mg/day)	37	23.8	1.6 (1.0 to 2.6)	0.075
	1 to 3 years (10 mg/day)	30	19.0	1.8 (1.1 to 3.1)	0.026
	3 to 10 years (10 mg/day)	45	25.5	3.0 (1.8 to 5.2)	<0.0001
	>10 years (10 mg/day)	9	24.3	3.7 (1.5 to 9.4)	0.0056
Current HCQ	No	95	29.0	1.0 (Ref. Gp)	
	Yes	90	14.6	0.5 (0.4 to 0.7)	<0.0001
HCQ use	Never	57	32.2	1.0 (Ref. Gp)	
	Past (not current)	27	27.6	0.9 (0.6 to 1.5)	0.81
	<6 consecutive months	16	20.0	0.6 (0.3 to 1.0)	0.056
	>6 consecutive months	64	13.8	0.5 (0.3 to 0.7)	0.0003
NSAID use	No	146	21.6	1.0 (Ref. Gp)	
	Yes	37	13.9	0.6 (0.4 to 0.9)	0.017
Aspirin use	No	137	17.9	1.0 (Ref. Gp)	
	Yes	49	28.0	1.6 (1.2 to 2.3)	0.0038

## Conclusion

For NCP, methotrexate increased NCP, possibly via homocysteine. For thrombosis, hydroxychloroquine significantly reduced thrombosis, but prednisone increased it. SLE longitudinal cohorts can address many clinical questions that are not suitable for clinical trials.

